# Genome-wide haplotype association study in imaging genetics using whole-brain sulcal openings of 16,304 UK Biobank subjects

**DOI:** 10.1038/s41431-021-00827-8

**Published:** 2021-03-04

**Authors:** Slim Karkar, Claire Dandine-Roulland, Jean-François Mangin, Yann Le Guen, Cathy Philippe, Jean-François Deleuze, Morgane Pierre-Jean, Edith Le Floch, Vincent Frouin

**Affiliations:** 1 Université Paris-Saclay, CEA, Neurospin, Gif-sur-Yvette, France; 2 Université Paris-Saclay, CEA, Centre National de Recherche en Génomique Humaine, Evry, France

**Keywords:** Genome-wide association studies, Magnetic resonance imaging, Genetics of the nervous system, Haplotypes, Predictive markers

## Abstract

Neuroimaging-genetics cohorts gather two types of data: brain imaging and genetic data. They allow the discovery of associations between genetic variants and brain imaging features. They are invaluable resources to study the influence of genetics and environment in the brain features variance observed in normal and pathological populations. This study presents a genome-wide haplotype analysis for 123 brain sulcus opening value (a measure of sulcal width) across the whole brain that include 16,304 subjects from UK Biobank. Using genetic maps, we defined 119,548 blocks of low recombination rate distributed along the 22 autosomal chromosomes and analyzed 1,051,316 haplotypes. To test associations between haplotypes and complex traits, we designed three statistical approaches. Two of them use a model that includes all the haplotypes for a single block, while the last approach considers each haplotype independently. All the statistics produced were assessed as rigorously as possible. Thanks to the rich imaging dataset at hand, we used resampling techniques to assess False Positive Rate for each statistical approach in a genome-wide and brain-wide context. The results on real data show that genome-wide haplotype analyses are more sensitive than single-SNP approach and account for local complex Linkage Disequilibrium (LD) structure, which makes genome-wide haplotype analysis an interesting and statistically sound alternative to the single-SNP counterpart.

## Introduction

Numerous population-imaging studies have been built since the beginning of years 2000 based on earlier pioneering studies [1] to support researches mainly in vascular, neurodegenerative diseases or psychiatric syndromes [2] and now include genetics [3–5]. Our work is based on the UK Biobank resources [6–8], currently the most emblematic imaging-genetics cohort available as open data. The UK Biobank cohort brings the unique opportunity to study the genetic and environmental dissection of numerous diseases or complex traits related to brain via imaging endophenotypes. These endophenotypes are structural or functional imaging-derived characteristics (or imaging-derived phenotypes—IDP [7] in UK Biobank) and DNA genotyping arrays provide the genetic measures [6]. In aging of normal and pathological brains, we observe the phenomenon of sulcal widening for which several related IDP like sulcal depth, sulcal opening and grey matter thickness in brain were shown by our group to be highly heritable [9]. In the case of grey matter thickness and sulcal opening, our group in [10] has also shown associations with several new markers using Genome-Wide Association Study (GWAS).

In order to yield robust inferences and interpretations from GWAS approaches, one should only consider the hits passing a strict genomic significance threshold [11] to account for the large number of tests. Moreover, in imaging-genetics studies, an additional correction for multiple phenotype testing is mandatory. After these corrections and in the case of a non-synonymous SNP hit, a straightforward interpretation can be carried out to hypothesise an association with the disease. In most cases, GWAS results are harder to interpret because the association is carried by a group of SNPs located in a non-coding region. In some cases, the grouping can be explained by a leading causal SNP, which signal is spread amongst the neighbouring SNPs via Linkage Desequilibrium (LD). Using imputed SNPs, one can identify putative unmeasured causal variants. Nowadays more and more GWAS are using imputed SNPs to perform fine-mapping or allow for replication study and meta-analyses. An alternative to imputation is haplotype analysis that can also capture unmeasured variants.

One might also suspect that the association is actually carried by more than one SNP in the same region. In the case of multiple causal SNPs, burden-test or collapsing test have shown a high power to detect small effect sizes [12]. A gain in sensitivity can also be obtained by considering the combinations of alleles from several genotyped SNPs in the form of haplotypes [13].

Haplotypes in one individual consist in the combinations of several SNP alleles to form nucleotide sequences. These sequences of alleles can be obtained from phased SNPs that define which alleles are on the same chromosome and inherited together from each parent.

Both phased SNPs and imputed variants are available in the UK Biobank resources, based on large reference panels (1000 Genomes and the Haplotype Reference Consortium (HRC), see [6]). Our method builds on the work done by UK Biobank to provide high quality phasing dataset (assessed using trios of parents-child), where the phase of individual SNPs can be used for haplotype association in a less computationally heavy framework than methods such as [14]. The method described in this paper differs from the commonly used haplotype-based approaches on several points. First, we investigate its behaviour on a genome-wide scale. Second, the haplotype-based tests rely on individual phased data rather than estimating haplotype frequencies, which is unusual: out of the 9 haplotype-based methods cited in [15], only one (WHaIT, [16]) uses phase information as input, and the others use an estimation of haplotype frequencies rather than individual phase data. Moreover, our method identifies individual haplotype associations by testing all haplotypes in a given genomic interval, while the WHaIT method only performs a global test, i.e. it does not identify which individual haplotype in the block is associated with the phenotype. Also of note is that the WHaIT method is only designed for categorical traits.

In this work, we propose to push the genome-wide haplotype association approaches to fit the specificity of the IDP obtained in imaging-genetics, more precisely sulcal opening measurements derived from the UK Biobank high-quality imaging data. The aim of the paper is three-fold. First, we present this set of quantitative IDP and a normalization of their distributions across the subjects. We show their spatial variability, which reveals that IDP are many and varied, and which motivates our search for even more sensitive association methods to compensate for the multiple testing issue. Second, we detail three tests for genome-wide haplotype associations with the traits. This part includes definition of blocks along the genome based on a genetic map, which is a prerequisite to any haplotype-based test definition. Third, we compare association hits obtained from these three haplotype-based tests and also with the regular genome-wide association test based on single SNPs. Finally, we carried out phenotype permutations to check the validity of our tests on the whole genome.

## Material and method

### Samples

The UK Biobank is a health research resource including genotypic data of about 500,000 people aged between 45 and 73 years old, that were recruited in the general population across United Kingdom. The original data considered in this study were obtained as part of the application #25251, which was approved by the UK Biobank ethics committee. The phenotypes computed from the original data were submitted to the UK Biobank Returns results catalogue and can be retrieved with reference to application id.

The UK Biobank is expected to provide multi-modal MR brain images in 100,000 participants and, by March 2018 it had 20,060 subjects with a T1-weighted MRI [8]. These data were processed locally through BrainVisa/Morphologist pipeline [17] and quality controlled yielding a set of labelled cortical sulci for each of 18,175 subjects (see [10] for details).

We relied on the Quality Control carried out by the UK Biobank consortium on the genotyping data which excluded individuals with high missingness, high heterozygosity, first degree related individuals or sex mismatches [6]. With 658,720 phased SNPs (Haplotype dataset), 784,256 genotyped SNPs and 93,095,623 imputed variants, 16,304 subjects identified as white British ancestry (as described in [6]) passed the image processing and the genetic QC (48% of males and 52% of females).

#### Replication sample

We processed subjects of non-white British ancestry and additional T1-weighted MRI images that were made available after the March 2018 release with BrainVisa/Morphologist sulci labelling pipeline. We obtained a replication dataset comprising 5070 individuals, 48% of males and 52% of females, 54% of white British ancestry.

### Data processing

#### Imaging data, sulcal opening

For each subject, 123 labelled sulci were extracted from T1-weighted images. For each sulcus, a measure of its width—a feature called opening—is computed as the ratio of the volume of the Cerebrospinal Fluid the sulcus contains to the surface of the sulcus (Supplementary Fig. 1).

For each sulcus, after adjusting for age and sex using linear regression, we identified and excluded outliers in the residual distribution using the robust interquartile range (IQR) method [18].

Then, since the distributions of sulcal opening values could exhibit deviation from the normal distribution, we evaluated this deviation and normalized them using a one-parameter Box-Cox transformation (power transformation) [19], see Supplementary Material 1. We selected the optimal power λ using goodness-of-fit for normal distribution (available in MASS R package) for each sulcus. The value of λ quantifies the similarity with a normal distribution. For a sulcus with an original normal distribution, λ = 1 and for a sulcus with an original log-normal distribution, λ = 0.

In the following, the Box-Cox transformations of the sulcal openings, Ỹ, were considered as phenotypes.

#### Definition of Haplotypes blocks in UK Biobank

When a group of SNPs are in linkage disequilibrium (LD), the assortment of their alleles is not random: the presence/absence of one allele at one locus depends on the alleles of neighbouring loci in LD. Using allele frequencies and LD information available in reference panels, the phasing process consists in estimating the sequence of SNP alleles that are on the same chromosome.

Using this approach, the UK Biobank consortium has released a curated genome-wide dataset of 658,720 phased SNPs over the 22 autosomal chromosomes [6]. The phasing was carried out using SHAPEIT [20] with the 1000 Genomes phase 3 dataset as reference panel. From these phased data we define haplotypes, which are combinations of neighbouring SNP alleles on a single chromosome. We propose the following twofold procedure to ensure the correct haplotype estimation.

First, we determine haplotype blocks that includes adjacent SNPs in high LD or in other words, present a low recombination rate. We used the GRCH37 genetic map that includes positions in base pairs (bp) and centiMorgans (cM) based on the 1000 Genomes project. For variants of the UK Biobank arrays that are not present in the genetic map, the position in cM was estimated using linear interpolation based on the local recombination rate (in cM/bp) within the interval defined by the two closest neighbouring variants in the map. For first (resp. last) assessed variants on a chromosome that fall outside the genetic map, recombination rates were estimated by linear extrapolation on the whole chromosome. The complete genetic map allows to define non-overlapping haplotype blocks in which any two consecutive SNP loci are less distant than δ cM. A distance greater than δ cM defines the start of a new block. Second, using the phased SNPs dataset and the blocks described above, we determine the haplotypes.

As a compromise between haplotype length and uncertainty, we chose δ value equal to 0.001 cM, leading to 119,548 haplotype blocks across the whole genome (see Supplementary Material 4 and [21]). For individual SNP phase, UK Biobank estimated a median switch error rate of 0.229% on chromosome 20 taken as an example [6], corresponding to a median value of 37 or 38 individual SNPs with phase errors. If all these errors were located in the 3048 haplotype blocks we obtained on that same chromosome, it would represent about 1% of errors in haplotype estimation, which is probably largely overestimated. In fact, we expect the error rate (or phase uncertainty) on haplotypes to be much less. The reason is the following: SNPs affected by a phase switch are most probably located in regions of high recombination rate, which are excluded from our haplotype blocks.

### Genome-wide haplotype association

#### Count matrix of each haplotype block

For each haplotype block obtained previously, we defined the reference haplotype *h*_**0**_ as the most common one, and the *count matrix:*

$${\mathbf{H}} = \left[ {h_1, \ldots ,h_m} \right]\,{\mathbf{ \in }}\,\left\{ {0,1,2} \right\}^{(N \times m)},$$

with *N* the sample size and *m* the number of alternative haplotypes denoted by ${h_1,...,h_m}$. Each element *hi, j* of **H** corresponds to the number of copies of the alternative haplotype ${h_j,{\mathrm{1}} \le j \le m}$ for the individual ${i,{\mathrm{1}} \le i \le N}$. In this way, the count matrix **H** codes for an additive haplotype model - the effect of each haplotype block corresponds to the sum of the effects of observed alternative haplotypes. Supplementary Material 2 shows an example of matrix **H**, obtained with 3 phased SNPs and 3 subjects

#### Association tests

We defined three association tests between haplotypes *h*_*j*_ included in matrix **H**, and the phenotypes ${\tilde Y}$, the sulcal opening values obtained after Box-Cox transformation as described previously. In the following, *X* denotes the matrix of covariates containing age, sex and the first ten principal components of genetic data provided by UK Biobank to account for population stratification. Although all presented methods are using similar statistics, the three tests are addressing three different questions.

##### Haplotype block model test

In this test, referred to as “block-test”, the following linear model is considered:

1 $${Y = \beta _0 + X\beta + H\gamma + \varepsilon }$$

with ${\tilde Y}$ the phenotype vector, *X* the matrix of covariates and **H** the matrix of the *m* alternative haplotypes, included with fixed effects *β* and *γ* respectively, and *ε* the error vector. *β*_0_ is the intercept containing the effect of the reference haplotype.

This first test assesses the association between a given block, defined as the set of its haplotypes, and each phenotype i.e. ${H_0:\gamma = 0_m}$ *vs*. ${H_1:{\mathbf{\exists }},1 \le i \le m\,\gamma _i \,\ne\, 0}$. The significance of the association is estimated using a total variance test for nested linear model.

#### Complete model individual haplotype test

The second test is a complete model individual haplotype test and is referred to as “complete-test”. It aims to test the association between one phenotype and each haplotype *h*_*j*_ inside the block, while considering in the model the other haplotypes of the block. For this purpose, we used the same linear model regression as in the block-test (Eq. 1) and considered for each haplotype *h*_*j*_, ${1 \le j \le m}$, the null hypothesis: ${H_0:\gamma _j = 0}$ *vs*. ${H_1:\gamma _j \,\ne\, 0}$. For this test, we computed a two-sided *p* value of the *t*-statistic.

#### Single haplotype model test

To test the effect of each haplotype versus the others, the third test referred to as “single-test” is based on the following linear model:

2 $${Y = \beta _0 + X\beta + h_j\gamma _j + \varepsilon \quad }{\mathrm{for}} \quad 0 \le j \le m$$

with ${\tilde Y}$ the phenotype vector, *X* the matrix of covariates and *h*_*j*_ the count vector for the haplotype, included with fixed effects *β* and *γ*_*j*_ respectively, and *ε* the error vector. *β*_0_ is the intercept containing the mean effect of all haplotypes excluding the considered haplotype *j*. As previously, we computed a standard two-sided *p* value of the *t*-statistic for the null hypothesis ${H_0:\gamma _j = 0}$ *vs*. ${H_1:\gamma _j \,\ne\, 0}$.

All three tests were implemented in R v.3.6.0 to run on a distributed HPC (code available upon request).

#### Comparison with single-SNP association used in classical GWAS

To our knowledge, a comprehensive power analysis of haplotype association for quantitative traits on large cohorts (more than 10,000 subjects) has not been done. To gain insight into the genome-wide significant *p* values yield by the haplotype association tests, we propose a comparative study, where we match the results of haplotype-based tests with the results from the single-SNP association used in classical GWAS considering both genotyped and imputed SNPs. The test used in classical GWAS using linear model in PLINK [22] is described in Supplementary Material 3.

#### Genome-wide significance threshold

##### Correction of *p* values and significance threshold for SNP associations

For single-SNP associations with imputed and genotyped SNPs, we used Bonferroni correction with the common estimated number of 10^6^ independent SNPs [23]. Furthermore, to account for testing 123 phenotypes, *p* values were corrected for *N*_GWAS_ = 123 × 10^6^ tests. Therefore, the threshold for genome-wide significance at risk α = 0.05 is

$${\mathrm{P}}_{{\mathrm{GWAS}}} = 0.05/{\mathrm{N}}_{{\mathrm{GWAS}}} = 4.0650407 \times 10^{ - 10}$$

##### Correction of *p* values and significance threshold for haplotype associations

For haplotype associations, we used Bonferroni correction for multiple testing, with N_T_ the number of hypotheses tested. For the n_b_ haplotype block tests, N_T_ ≈ 123 × n_b_. For the *n*_h_ individual haplotype (complete or single) tests, N_T_ ≈ 123 × n_h_. The genome-wide significance threshold at risk α = 0.05 is P_NT_ = α ∕ N_T_ that is: P_NT_ = α **∕**14,703,239 = 3.4 × 10^−9^ for the block-test; P_NT_ = α **∕**107,904,126 = 4.63 × 10^−10^ for the complete-test and P_NT_ = α **∕**125,218,783 = 3.993 × 10^−10^ for the single-test.

#### False positive rates in the genome-wide haplotype association tests

In order to evaluate the validity of the different haplotype association tests scrutinized in this study, we constructed datasets under the null hypothesis using permuted phenotypes. Then, we computed the *p* values distributions and the associated False Positive Rate (FPR). The FPR is the percentage of tests that show a significant association in the permuted dataset at the significance threshold.

Correlations between variables are present in genetic and imaging datasets; between parts of the genome and within the brain respectively. To quantify the impact of these correlations, we computed the FPR under null hypothesis for several scenarios that keep or not the correlation structures among haplotype blocks and among phenotypes.

##### False positive rate while preserving the correlation within each haplotype block

In the first permutation scenario, a phenotype is randomly permuted for each block. Therefore, correlations between haplotype blocks across the genome present in the original dataset were removed in this scenario, preserving only the correlation within each haplotype block. The complete-test and the single-test produced several statistics per haplotype block (depending on the number of haplotypes), while the block-test produced a unique statistic per block. We computed ${FPR_p = N_{FP}/N_{t,p}}$ where *N*_FP_ is the observed number of false positives and ${N_{t,p}}$is the number of statistics produced by each of the three tests for the permuted phenotype *p*.

This permutation scenario was replicated on three phenotypes of sulcal opening: two that represent the range of λ values and one for the highest association found in previous study [10], with λ values ≈0.2, ≈0.8 and ≈0.6, respectively. The same permutation order was applied to the three phenotypes.

##### False positive rate while preserving the correlation within the genome

In the second permutation scenario, we used all the phenotypes and produced a permutated dataset where the phenotypes were randomly shuffled with the same permutation for all haplotype blocks along the genome. For each of the three tests, we considered all the statistics produced and computed ${FPR_p = N_{FP}/N_{t,p}}$ where *Nt, p* is the number of statistics produced for a given permutated phenotype *p*. This analysis produced three FPRs per permuted phenotype that preserves the structure of the correlation within the genome. Reporting FPRs for each phenotype will enable the detection of phenotypes with low-quality measurements.

This procedure was replicated 25 times. To reduce the computational burden in this case, we considered the residuals ${\tilde Y^ \ast = \tilde Y - \left( {\widehat \beta _0 + X\widehat \beta } \right)}$, that is, the phenotypes ${\tilde Y}$ adjusted for the covariates

##### False positive rate while preserving the correlation within the genome and among the phenotypes

Last, we used the permutated dataset preserving the correlation within the genome and, instead of considering each phenotype independently, we pooled the results across the phenotypes. For each permutation and each haplotype test, we considered all the statistics produced. We computed *FPR*_*T*_ = *N*_*FP*_ / *N*_*T*_ where *N*_*T*_is the number of statistics produced by each test *for all permutated phenotypes*. This analysis produced three FPRs per permutation, that preserve both the structure of the correlation within the genome as well as the correlation among phenotypes.

## Results

In this part, we present results obtained on real data, the opening measurements of 123 brain sulci and genome-wide haplotypes of 16,304 subjects. First, we present the effects of normalization of the phenotype distributions, and the characteristics of the haplotypes generated with the phased data provided by UK Biobank. Second, we present the significant association hits obtained with our genome-wide haplotype association study and provide the genomic location and length of significant haplotype blocks. Finally, we compare the differences in sensitivity on real data for the three tests proposed above and we propose a comparison study with the single-SNP association test.

A study of the False Positive Rate is also presented, where we study the FPR for the three models under the null hypothesis, using permutated datasets as described in section “False Positive Rates in the Genome-wide haplotype association tests”.

### Imaging data processing

**Fig. 1:**
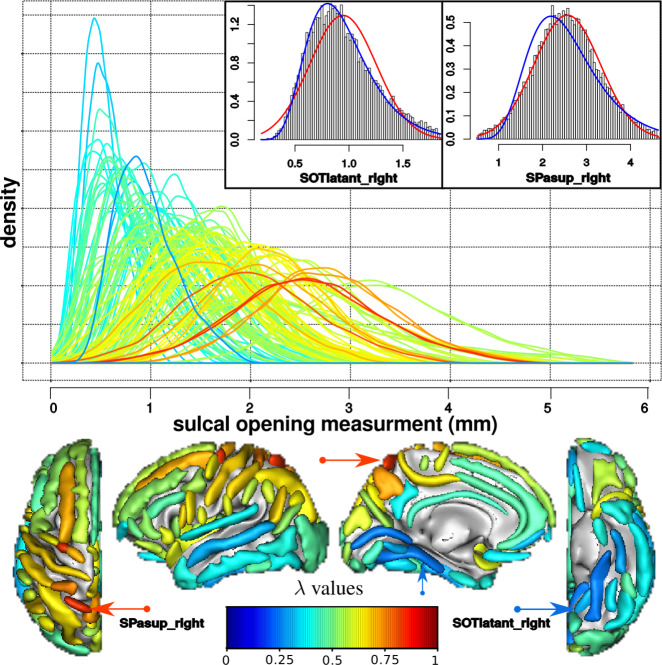
In a sample of 16, 304 subjects, sulcal opening measurements have different distributions across the brain, from normal distribution (red, λ = 1) to log-normal (blue, λ = 0). (Top) Distributions of uncorrected sulcal opening measurements, for 123 sulci, coloured according to λ values. Inlet panels are histograms of opening values for two sulci with fitted curves superimposed: (left) Anterior occipito-temporal sulcal opening distribution is almost log-normal (blue line); (right) Superior parietal sulcal opening distribution is almost normal (red line). (Bottom) Spatial map of λ values for sulci of the left hemisphere, coloured according to the λ value of their distribution. From left to right: top view, interior view, parietal view and bottom view.

The distributions of sulcal opening measurements across the brain exhibit a large range of density shapes from quasi-normal distributions (max. λ = 0.8) to log-normal distributions (min λ = 0.2). Figure 1 displays the λ parameter associated with the opening distribution for each sulcus across the brain. The spatial distribution of the λ parameter across the brain highlights the necessity of such a transformation.

We observed a pattern, with larger (in terms of median value) and more normally distributed sulcal opening measurements in parietal and occipital lobes. Conversely, we observed narrower, more log-normally distributed opening values for lower and inferior temporal sulci. This could be related to the differential aging rate of brain structures, such as the widening sulci rates that might differ across the brain [24]. Based on the results from FPR study, we show that this procedure ensures a relatively homogeneous specificity of the analysis across the brain sulci.

### Genomic data processing

**Fig. 2:**
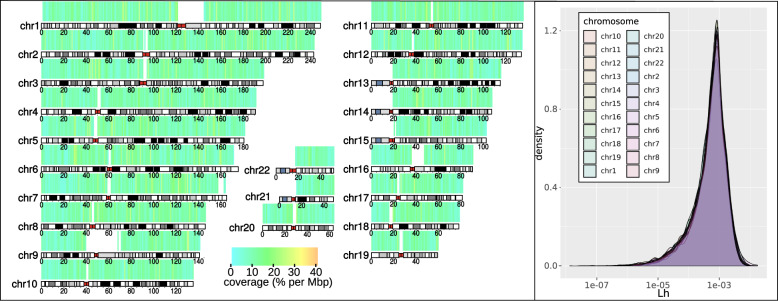
(Left) Genome coverage of haplotype blocks, in percent per Mbp. For each window of 1 Mbp, we computed the total length in bp covered by the 119,912 haplotype blocks (see section “Data processing” for details). (Right) Distributions of L(h) (block length in cM), as defined by the genetic distance between bordering markers of each block. Blocks were defined over the 22 autosomal chromosomes using δ = 0.001 cM, a parameter that account for the maximal length in cM between two consecutive markers in a same haplotype (see section “Data processing” and [21] for details). The distribution of block lengths is homogeneous across the 22 autosomal chromosomes.

Across the 22 autosomal chromosomes, we tested over 1 million haplotypes from 119,548 blocks for association with the opening of 123 sulci. Candidate haplotypes had a length, on the genetic map, ranging from 1.4 × 10^−8^ to 1.7 × 10^−2^ cM (see Fig. 2, right panel), with a median value of 6 × 10^−4^ cM, corresponding to consecutive SNP runs of length ranging from 2 to 64, with an average of 7.498 haplotypes per block (min. 2, max. 1588, median 3), including the major haplotype. Left panel of Fig. 2 shows the coverage of the blocks along the 22 autosomal chromosomes. Details regarding the distribution can be found in Supplementary Material 4. Of note is that the variation of the block length across the chromosomes remains small as it can be seen from the curve on the right of Fig. 2 (similar distributions).

### Sulcal opening patterns are associated with haplotypes

In this study we show significant associations of sulcal opening measurements with haplotypes in a genome-wide approach; bringing about genome-wide inferences that differ from a local haplotype fine-mapping operation performed after a classical genome-wide single-SNP analysis. Along with the previous findings in the upstream region of KCNK2 (chr1:215MB) [10], the haplotype block test revealed four other hits on chromosomes 7, 9, 12 and 16, detailed in Table 1. For the following genes, located in these regions, that are predominantly expressed in the brain: LPAR1 (chr9); NUAK1 (chr12), lncRNA RP11-178L8.8 and FBXO31 (chr16), we provide an overview of expression data, publicly available, in Supplementary Material 6. We also display brain regions associated with each hit in Supplementary Material 9. Except for chr9:114, all hits reported on Table 2 are associated bilaterally with sulci such as (FCMpost_L, FCMpost_R) for chr1:215, (STsterascant_R, STsterascpost_L) for chr12:106 and (SFinfant_L, SFinfant_R) for chr16:87.

**Table 1:** Genes or regions of interest related to haplotypes significantly associated with the opening of at least one sulcus. All positions are given for GRCh37/hg19 assembly.

Haplotype position	Gene / Region	Description
chr1: 215146807- 215154276	upstream of KCNK2	eQTL of KCNK2 [10]
chr7: 13441632- 138543686	chr7: 134346283- 134446935	autism susceptibility region7q32.3-q33 [24]
	chr7: 129202420- 138543686	AUTS1 region: D7S530 to D7S684 [25, 26]
chr9: 113659382- 113665419	chr9: 113635543- 113800981 (intronic)	*LPAR1: Homo sapiens lysophosphatidic acid receptor 1, transcript variant 2, mRNA. Protein, most expressed in Brain - Spinal cord (cervical c-1) (GTEx V7)*
chr12: 106476140- 106477376	chr12: 106457118- 106533811 (intron, exon 3 & 4)	NUAK1: Homo sapiens NUAK family, SNF1-like kinase, 1, mRNA. Protein, most expressed in Brain - Frontal Cortex (BA9) (GTEx V7), involved in axon branching [32]
chr16: 87226206- 87257820	chr16: 87360593- 87361190 (102kpb, upstream)	*RP11-178L8.8 (AC010531.7): novel transcript, antisense to FBXO31 and C16orf95 readthrough LncRNA, most expressed in Brain - Cerebellum(GTEx V6)*
	chr16: (102kpb, upstream) 87360593- 87422364	*FBXO31: F-box protein 31,mRNA. Protein, most expressed in Brain - Cerebellum(GTEx V8)*

**Table 2:** Significant hits found in haplotype blocks.

chr	Start (bp)	Length (kbp)	Phenotype	Haplotype tests (corrected *p* values)			SNP tests (corrected *p* values)			
				Block	Complete	Single	Genotyped SNPs	Imputed SNPs	Rare SNPs	Min (All SNPs)
1	215,146,807	7.469	FCMpost_L	**7.30E−17**	1.45E−13	6.79E−12	2.47E−10	3.38E−11	n.s.	3.38E−11
			FCMpost_R	**1.37E−14**	1.36E−12	1.65E−09	4.94E−11	5.81E−12	n.s.	5.81E−12
			FIP_L	n.s.	**7.87E**−**04**	n.s.	n.s.	n.s.	n.s.	n.s.
			FIP_R	1.35E−03	**2.44E**−**05**	5.64E−04	5.45E−04	5.61E−04	n.s.	5.45E−04
			FPO_R	3.77E−03	**4.12E**−**06**	5.33E−05	3.26E−05	4.40E−05	n.s.	3.26E−05
			SC_R	n.s.	**2.86E**−**05**	3.44E−03	3.08E−03	3.15E−03	n.s.	3.08E−03
			SFint_L	n.s.	**4.98E**−**02**	n.s.	n.s.	n.s.	n.s.	n.s.
			SFsup_R	n.s.	**1.08E**−**02**	n.s.	n.s.	n.s.	n.s.	n.s.
			SPaint_L	1.89E−04	**5.72E**−**05**	n.s.	n.s.	n.s.	n.s.	n.s.
			SpC_L	1.09E−04	**8.12E**−**07**	1.95E−03	1.09E−03	1.09E−03	n.s.	1.09E−03
			SsP_L	3.51E−03	**2.97E**−**06**	4.03E−03	2.20E−03	3.55E−03	n.s.	2.20E−03
7	134,416,326	0.278	INSULA_L	**1.22E**−**02**	n.s.	n.s.	n.s.	n.s.	n.s.	n.s.
			INSULA_R	**2.61E**−**02**	n.s.	4.21E−02	n.s	4.09E−02	n.s.	4.09E−02
9	113,659,382	6.037	FCMpost_R	**9.55E**−**03**	4.14E−02	3.43E−02	1.94E−02	1.57E−02	n.s.	1.57E−02
			SPaint_L	n.s.	3.33E−02	2.84E−02	3.61E−02	2.83E−02	n.s.	2.83E−02
12	106,476,140	1.236	FCLp_R	n.s.	5.10E−04	**9.41E**−**05**	1.44E−04	1.02E−04	n.s.	1.02E−04
			SFint_R	4.25E−04	9.22E−05	**8.20E**−**06**	4.56E−05	1.14E−05	n.s.	1.14E−05
			SPeCinter_L	n.s.	n.s.	1.29E−02	2.50E−02	1.37E−02	n.s.	1.37E−02
			STsterascant_R	n.s.	1.33E−02	2.40E−03	**6.25E**−**04**	1.59E−03	n.s.	6.25E−04
			STsterascpost_L	n.s.	n.s.	**1.81E**−**02**	2.28E−02	2.35E−02	n.s.	2.28E−02
16	87,226,206	2.984	SFinf_L	2.01E−03	1.28E−04	2.55E−05	1.50E−05	**1.46E**−**05**	n.s.	1.46E−05
			SFinfant_L	n.s	n.s	3.14E−02	1.68E−02	2.44E−02	n.s.	1.68E−02
			SFinfant_R	2.04E−06	1.00E−06	7.03E−09	**1.16E**−**09**	4.25E−09	n.s.	1.16E−09
			SFinter_R	3.36E−02	1.36E−02	1.37E−03	9.94E−04	**7.28E**−**04**	n.s.	7.28E−04
			SFmarginal_L	n.s.	**1.05E**−**02**	3.13E−02	2.67E−02	4.23E−02	n.s.	2.67E−02
16	87,245,155	1.453	SFinf_L	8.33E−04	5.96E−03	1.65E−04	**9.53E**−**05**	1.76E−04	n.s.	9.53E−05
16	87,254,180	3.64	SFinf_L	1.31E−03	3.76E−02	n.s	6.59E−04	**4.55E**−**04**	n.s.	4.55E−04
			SFinfant_L	n.s.	5.42E−03	5.39E−03	1.47E−03	**1.20E**−**03**	n.s.	1.20E−03
			SFinfant_R	1.80E−02	5.81E−03	5.20E−03	2.18E−03	**1.12E**−**03**	n.s.	1.12E−03

For each block, the lowest *p* value over the three haplotype tests is compared with the lowest *p* value in the block for genotyped SNPs, imputed common SNPs (MAF < 1%) and rare SNPs (MAF > 1%). For each row, the lowest *p* value is highlighted in bold. All positions are given for GRCh37/hg19 assembly. All *p* values are adjusted using Bonferroni corrections. Association yielding *p* values that did not pass the genome-wide threshold are noted as *n.s*. and bold numbers indicate lowest *p* values observed for each row.

The bilateral associations of the insula (left and right) in chr7:134 were not found on the replication study. However, it stands out since the associated brain areas are not associated with any other genome region. Further studies could be motivated to investigate this genomic region since the insula is a cortical region known to integrates emotional, cognitive, and motivational signals, and that haplotypes of the block chr7:13441632-134416604 are located in a region carrying several genes and markers associated with Autism Spectrum disorders ([25–27], see details in Table 1).

### Haplotype tests exhibit different sensitivity compared to single SNP-based associations

**Fig. 3:**
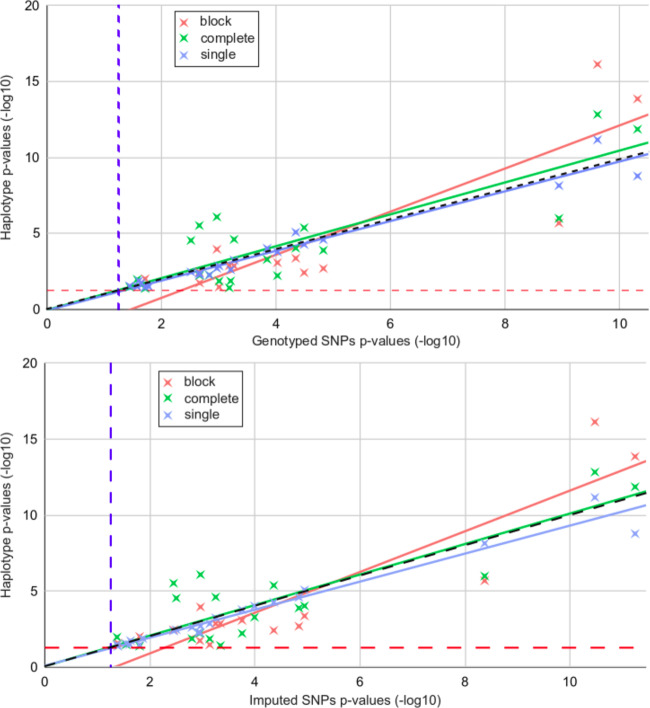
Comparison for main hits using the three proposed haplotype association tests (*Y*-axis) and single-SNP association (PLINK, *X*-axis) with: (top) genotyped SNPs and (bottom) imputed SNPs. For each significant haplotype-phenotype association (*Y*-axis), the lowest *p* value observed for the SNPs inside the block was reported (*X*-axis). For points above the grey line at x = y, *p* values for haplotype associations test are lower than their single-SNPs counterpart. Red lines indicate the fit of haplotype block test *p* values (*y* = 1.34 *x* + −1.77, *R*^2^ = 0.827) with imputed SNPs and (*y* = 1.42 *x* + −2.07, *R*^2^ = 0.765) with genotyped SNPs; Green lines indicate the fit of complete model individual haplotype test *p* values (*y* = 1 *x* + 0.0716, *R*^2^ = 0.744) with imputed SNPs and (1.05 *x* + −0.031, *R*^2^ = 0.709) with genotyped SNPs; Blue lines indicates the fit for single haplotype test *p* values (*y* = 0.922 *x* + 0.099, *R*^2^ = 0.959) with imputed SNPs and (*y* = 0.98 *x* + −0.0531, *R*^2^ = 0.951) with genotyped SNPs. Vertical, blue dashed lines indicate genome-wide significant threshold for SNPs, Horizontal, red dashed lines indicate genome-wide significant threshold for haplotype associations. All *p* values are corrected using Bonferroni correction (see details in section “Genome-wide significance threshold”).

We carried out a comparative study of the genome-wide significant *p* values yield by the three haplotype tests. To do so, all significant phenotype-haplotype associations for the three tests were matched with the conventional SNP association results. In Fig. 3 top panel, the hits obtained with the three tests were matched with genotyped SNPs and with the imputed SNPs in the bottom panel of Fig. 3. For each significant phenotype-haplotype association, we used the lowest *p* value of single-SNP associations observed in the SNPs of that haplotype block. For the complete-test and the single-test, if more than one haplotype per block was significantly associated, we selected the one with the lowest *p* value. When comparing results among the three tests, we found that:

(i) the block-test, that combines all haplotypes present in one block, leads to the lowest *p* values for the strongest associations. However it appears globally more conservative than the single-SNP approach, particularly for associations in a range close to the genomic threshold (10^−2^ to 10^−4^, corrected). The negative intercept of the fitted linear trend (red lines in Fig. 3) indicates that, in the significant blocks, *p* values from this test are on average less significant than their single-SNP counterparts.
(ii) the complete-test shows the highest sensitivity for moderate associations with some *p* values being more than 3 orders of magnitude lower than their single-SNP counterparts (resp. 8.121 × 10^−07^ and 1.09 × 10^−03^).
(iii) the single-test performs similarly to the single-SNP approach, with *p* values being slightly closer to those obtained with genotyped SNPs (*R*^2^ = 0.959) compared to those obtained with imputed SNPs (*R*^2^ = 0.951), while the opposite was observed for block and complete test. Indeed, a large number of haplotypes are differentiated from the other haplotypes by a single SNP in the same block, and thus are expected to carry the same signal.

In Table 2 we used three available SNP datasets: (a; genotyped SNPs): SNPs called from the UK-Biobank arrays (no MAF filtering); (b; Imputed SNPs): imputed SNPs see [6] with a MAF > 0.01; (c; Rare SNPs): imputed SNPs with MAF < 0.01. All MAF were computed using –maf options in PLINK, joining discovery, and replication dataset. Similarly to Fig. 3, in order to match a given haplotype hit with a single-SNP hit in Table 2, we used the lowest *p* value of single-SNP associations observed in the SNPs of that significant haplotype block.

The comparative analysis given in Table 2 revealed the following salient points:

(i) Haplotype association tests yield similar or better *p* values than imputed or genotyped SNP counterparts. Indeed, in the majority of hits, there was at least one haplotype model that was more significant than the single-SNP test (19 vs. 10). Moreover, for six phenotype/genotype significant associations, haplotype *p* values were at least 10-fold lower than their single-SNP counterparts while the opposite was not observed.
(ii) For strong associations involving several haplotypes in the same block, haplotype approaches can lead to *p* values that are several order lower, for example, haplotypes association in block chr1:215146807-215154276 with FCMpost Left leads to corrected *p* values in the range of 10^−17^ compared to 10^−11^ with single-SNP associations (resp. (10^−24^ and 10^−19^ uncorrected). Additionally, for 5 other phenotypes, *p* values obtained using haplotype associations are also 2 or 3 orders of magnitude lower (6 phenotypes for uncorrected values).
(iii) Imputed rare variants do not exhibit any significant associations, so we did not run any test on rare variants in the replication dataset. Indeed, the smallest uncorrected *p* values associated with rare variants are 6 to 20 orders of magnitude higher than the significant haplotype hits (data not shown), indicating a potential lack of power given the sample size.

### False positive rates in the Genome-wide haplotype association tests

In Supplementary Material 5, we investigated FPR in the two first scenarios of null hypotheses as described in section “False positive rates in the Genome-wide haplotype association tests” and reported results in Supplementary Fig. 1 (for scenario 1) and Supplementary Table 1 (for scenario 2). We show that in the second permutation scenario, the three tests seem to control the family-wise error rate for most phenotypes, as well as on average overall phenotypes. The spatial pattern in the λ values does not reflect the spatial distribution of the FPR across the brain given in Supplementary Table 1 (*t*-test with *p* values > 0.05 for all three haplotype testing strategies, using Pearson’s correlation moment between all phenotypes’ λ values and average FPR) and we observed an overall homogeneous behaviour across the brain for the three haplotype tests. Moreover, out of the two phenotypes (FCalant.ScCal_left and SRh_left) showing systematic inflated FPR, none of them were involved in significant hits on real data. In Supplementary Fig. 4, we did not observe any inflation of FPR under the first permutation scenario. In addition, we did not observe any false positive.

**Fig. 4:**
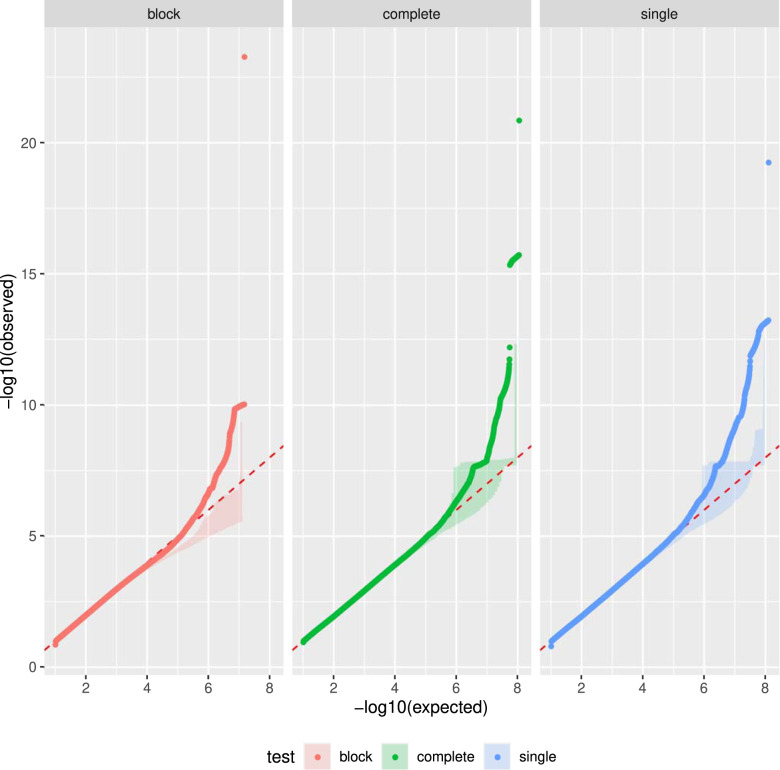
Aggregation of Q–Q plots for 25 runs of permutated datasets (see section “False positive rates in the Genome-wide haplotype association tests”, third scenario). From left to right: block-test, complete-test and single-test. The shaded area define the hull (minimum and maximum values) of the Q–Q plots for the 25 permutated datasets and the Q–Q plot obtained on real data is given by the coloured, dotted line. The red, dashed line at *y* = *x* indicates where the expected distribution lies.

Table 3 shows an estimation, for each test, of FPR_T_ = N_FP_ / N_T_, the FPR for the null distributions that preserve the correlation among phenotypes and within the genome (scenario 3 described in section “False positive rates in the Genome-wide haplotype association tests”). When using Bonferroni correction for significance thresholds (see section “False positive rates in the Genome-wide haplotype association tests”), only the block test seems to control for Family-Wise Error Rate, while the complete and single-tests show an inflation of FPR (see Table 3). The Q-Q plots presented in Fig. 4 indicate that in this permutation scenario, the inflation of FPR was caused by extreme *p* values (probably due the two phenotypes with an FPR inflation in the permutation scenario by phenotype) rather than a systematic inflation of the statistic. In order to identify the phenotypes associated with these extreme *p* values, we first removed phenotypes for which more than 25% of individuals are missing but this did not reduce FPR inflation (see Supplementary Table 2 and Supplementary Fig. 5). Then we also removed the two phenotypes with an FPR inflation, which allowed us to control the Family-Wise Error rate (see Supplementary Table 2 and Supplementary Fig. 6).

**Table 3:** False positive rate under null hypothesis for 25 runs of permutations using the third scenario (see section “False positive rates in the Genome-wide haplotype association tests”).

Test	Average number of tests per run	Average discovery threshold α′=α/NT	Average FPR per run	Average # FP per run	# of runs	# of runs with FPR ≥ 1/N_T_	proportion of runs with FPR ≥ 1/N_T_	Min FPR	Max FPR
block	14,700,330	3.40E−09	2.72E−09	0.04	25	1	0.04	0	6.80E−08
complete	110,575,583	4.52E−10	1.81E−09	0.2	25	5	0.2	0	9.06E−09
single	124,659,108	4.01E−10	9.59E−10	0.12	25	3	0.12	0	8.00E−09

For each run of permutation, tests were corrected by the Bonferroni procedure P_T_ = α ∕N_T_ for N_T_ tests with risk α = 0.05 (see section “Genome-wide significance threshold”). The FPR should be lower than 1/N_T_ in 5% of the runs in absence of inflation.

### Significant haplotype associations are found in replication dataset

For all significant associations, we conducted a replication study and, with the exception of the hits on block chr7:134416326-134416604, all hit locations were found significantly associated in the replication study (see Table 4). However, the replication rate of haplotype associations was average for the block test (58%), and high for complete and single-tests (resp. 91% and 86%). Overall single-SNP test hits were less frequently replicated than hits from haplotype tests. For the haplotype block model test, out of 13 phenotypes significantly associated in the original dataset, 7 (53%) were found significant in the replication study. This ratio was comparable in Imputed SNPs and Genotyped SNPs (resp. 45 and 57%) but well below single-test and complete haplotype test (resp. 84 and 94%).

**Table 4:** Replication study for haplotype and SNP hits.

chr	Start (bp)	Phenotype	Block		Complete		Single		Genotyped SNPs		Imputed SNPs	
			Disc.	Rep.	Disc.	Rep.	Disc.	Rep.	Disc.	Rep.	Disc.	Rep.
1	215,146,807	FCMpost_L	7.30E−17	1.25E−04	1.45E−13	6.26E−09	6.79E−12	9.23E−08	2.47E−10	3.27E−06	1.43E−11	5.54E−06
		FCMpost_R	1.37E−14	2.57E−04	1.36E−12	1.79E−07	1.65E−09	1.34E−05	4.94E−11	3.48E−04	1.46E−12	1.24E−03
		FIP_L	n.s.	n.t.	7.87E−04	1.88E−02	n.s.	n.t.	n.s.	n.t.	n.s.	n.t.
		FIP_R	1.35E−03	n.s.	2.44E−05	1.04E−02	5.64E−04	8.22E−03	5.45E−04	n.s.	4.94E−04	n.s.
		FPO_R	3.77E−03	n.s.	4.12E−06	n.s.	5.33E−05		3.26E−05	n.s.	3.65E−05	n.s.
		SC_R	n.s.	n.t.	2.86E−05	6.46E−04	3.44E−03	8.01E−04	3.08E−03	2.40E−02	3.15E−03	4.86E−02
		SFint_L	n.s.	n.t.	4.98E−02	8.66E−03	n.s.	n.t.	n.s.	n.t.	n.s.	n.t.
		SFsup_R	n.s.	n.t.	1.08E−02	1.69E−04	n.s.	n.t.	n.s.	n.t.	n.s.	n.t.
		SPaint_L	1.89E−04	1.24E−02	5.72E−05	2.31E−03	n.s.	n.t.	n.s.	n.t.	n.s.	n.t.
		SpC_L	1.09E−04	n.s.	8.12E−07	4.51E−02	1.95E−03	n.s.	1.09E−03	n.s.	8.07E−04	n.s.
		SsP_L	3.51E−03	n.s.	2.97E−06	2.45E−02	4.03E−03	1.34E−02	2.20E−03	n.s.	3.22E−03	n.s.
7	134,416,326	INSULA_L	1.22E−02	n.s.	n.s.	n.t.	n.s.	n.t.	n.s.	n.t.	4.09E−02	n.s.
		INSULA_R	2.61E−02	n.s.	n.s.	n.t.	4.21E−02	n.s.	1.94E−02	7.76E−03	1.57E−02	1.98E−02
9	113,659,382	FCMpost_R	9.55E−03	2.91E−03	4.14E−02	2.33E−04	3.43E−02	2.58E−04	3.61E−02	n.s.	2.83E−02	n.s.
		SPaint_L	n.s.	n.t.	3.33E−02	2.62E−03	2.84E−02	3.38E−03	1.44E−04	n.s.	1.02E−04	n.s.
12	106,476,140	FCLp_R	n.s.	n.t.	5.10E−04	6.18E−03	9.41E−05	4.67E−03	4.56E−05	2.68E−03	1.14E−05	1.80E−02
		SFint_R	4.25E−04	3.10E−03	9.22E−05	4.43E−04	8.20E−06	1.92E−04	n.s.	n.t.	n.s.	n.t.
		SPeCinter_L	n.s.	n.t.	n.s.	n.t.	1.29E−02	7.44E−03	2.50E−02	n.s.	1.37E−02	n.s.
		STsterascant_R	n.s.	n.t.	1.33E−02	7.65E−04	2.40E−03	3.25E−04	6.25E−04	5.26E−03	1.59E−03	3.05E−02
		STsterascpost_L	n.s.	n.t.	n.s.	n.t.	1.81E−02	n.s.	2.28E−02	2.68E−03	2.35E−02	1.55E−02
16	87,226,206	SFinf_L	2.01E−03	1.25E−03	1.28E−04	2.73E−04	2.55E−05	2.71E−05	1.50E−05	5.95E−04	7.84E−06	1.44E−03
		SFinfant_L	n.s.	n.t.	n.s.	n.t.	3.14E−02	7.07E−05	1.68E−02	1.99E−03	2.44E−02	2.89E−04
		SFinfant_R	2.04E−06	n.s.	1.00E−06	1.01E−02	7.03E−09	1.05E−02	1.16E−09	n.s.	4.25E−09	n.s.
		SFinter_R	3.36E−02	2.16E−03	1.36E−02	8.46E−05	1.37E−03	3.11E−05	9.94E−04	5.90E−04	4.23E−05	2.92E−04
		SFmarginal_L	n.s.	n.t.	1.05E−02	8.78E−04	3.13E−02	2.50E−04	2.67E−02	3.54E−03	4.23E−02	1.69E−02
16	87,245,155	SFinf_L	8.33E−04	5.82E−04	5.96E−03	n.t.	1.65E−04	4.01E−03	9.53E−05	n.s.	1.07E−04	n.s.
16	87,254,180	SFinf_L	1.31E−03	2.94E−06	3.76E−02	1.13E−05	n.s.	n.t.	6.59E−04	2.26E−04	4.55E−04	2.87E−04
		SFinfant_L	n.s.	n.t.	5.42E−03	8.45E−04	5.39E−03	1.09E−03	1.47E−03	4.86E−03	1.20E−03	1.54E−02
		SFinfant_R	1.80E−02	1.21E−03	5.81E−03	5.83E−05	5.20E−03	4.71E−05	2.18E−03	1.03E−03	1.12E−03	4.28E−03
Total Hits			17	10 (58.82%)	24	22 (91.67%)	23	20 (86.96%)	25	14 (56.%)	32	14 (43.7%)
Total Phenotypes			13	7 (53.85%)	19	18 (94.74%)	19	16 (84.21%)	19	11 (57.9%)	24	11 (45.8%)

All replication *p* values were adjusted using Bonferroni correction for the number of significant associations in the original dataset. Associations that were not significant are displayed as (n.s). Associations that were not tested in the replication dataset are displayed as (n.t.).

## Discussion

### Choosing δ value to define blocks

We studied a range of values for the δ parameter from 10^−3^ to 2.5 10^−2^ cM, that balance (i) the size of haplotype blocks, by including SNPs further apart on the map, while ensuring (ii) the reliability of the phase along the block [21]. We set the δ parameter to the smallest value (smallest probability of recombination). Of note, we observed that the distribution of the block genetic length L(h) for this value is the most homogeneous across the different chromosomes. In addition, in Supplementary Material 4 we show that with δ increasing from 0.001 to 0.025, the distribution becomes bi-modal. Moreover, with a higher δ value, a larger number of recombination events is allowed within the same haplotype block which may lead to a lower sensitivity. One could see our dataset as a good representation of the UK Biobank overall dataset and for any other representative subsets of UK Biobank or genetic datasets based on the same panel and genetic map, we could recommend using the same value. Moreover, for genetic datasets of the same quality and based on the same or related panel and genetic map (namely 1000 Genome Phase 3 or HRC panel), the FPR study suggests that one can safely use our method on several related phenotypes. For genetic datasets based on another panel that might harbour different correlation structures across the genome, or phenotypes that follow particular distributions, one might need to perform a new FPR study using permutations such as described in this work.

### Recommended strategy for genome-wide haplotype associations in imaging genetics study

When applied on a single phenotype (second permutation strategy), the three tests showed control of FPR for most phenotypes (see Supplementary Table 1). However a few phenotypes may lead to abnormally low *p* values, especially with the complete and the single-tests, that even resist the Bonferroni correction applied in the third permutation strategy (when all phenotypes are tested at the same time). In this third scenario, only the block test controls the family-wise error rate.

Our results on real data show that in the presence of a complex association signal, like in the region upstream of KCNK2, the haplotype modelling underlying the block-test model produces *p* values several orders of magnitude lower. This result calls for the development of innovative multivariate approaches based on haplotypes, such as those the authors have proposed in [28].

In our case, the block on Chr1:215 harbouring the strongest signal is also the block with the most complex associations (with several significant SNPs, see [10]). However one could expect complex signals currently lying below the GWAS detection threshold to be picked up by the haplotype block test. Individual haplotype tests (single and complete tests) could be ran in a second step to identify haplotypes of interest inside the significant blocks.

### Limitations of the study

We choose to use a low δ value in order to obtain the most reliable haplotypes. One drawback is that this also allows higher LD between the blocks, meaning a greater correlation between blocks than with higher δ values. Using Bonferroni correction for multiple testing is expected to be conservative in the presence of such dependencies. In this situation, Howard et al. [29] proposed a correction using an estimation of the number of independent haplotypes, which would be less stringent than the Bonferroni correction.

In our study of FPR, we purposely focus on realistic null distributions of measurements, that exhibit the true correlation *between all the phenotypes and along the whole genome*. To have a better estimation of the FPR, one would need to run a larger number of runs which is computationally intensive and out of reach using our realistic null distributions. However, with 25 permutations used, we still could reasonably detect when an inflation of FPR occurs.

By comparing with single-SNP analyses, we did not estimate the discovery power of our tests. To our knowledge, for whole-genome haplotype associations, there is no practical way to create a synthetic and realistic ground truth signal for multiple correlated phenotypes.

### Conclusion and future works

In the context of imaging-genetics, we studied three haplotype tests on a genome-wide and phenotype-wide approach to find associations between haplotypes and quantitative traits measured on brain MRI. Beyond the process of extracting features from the imaging data, we normalized the measurement distributions of the sulcal openings using Box-Cox transformations and obtained spatially homogeneous distributions across the brain. We compared three haplotype association tests and achieved the best performance with the haplotype block model test (lowest *p* values observed and best control of FPR). The complete model individual haplotype test can then be used to identify the haplotypes of interest within the associated block. These two tests use a multivariate model of haplotypes that accounts for the whole set of haplotypes occurring in the studied population for a given block. Based on the results of this work, we do not argue for the systematic use of haplotype modelling over classical GWAS based on imputed variants. More particularly, in the case of the UK Biobank, all samples come from a homogeneous population with an extensive imputation panel [6] and haplotype association tests often yield *p* values in the same range as that of imputed SNPs. However, in case of complex signals, we argue that the haplotype block test could provide a complementary approach that detects associations that lie below GWAS discovery threshold. Moreover haplotype associations seem to be easier to replicate than single-SNP associations. This study relies on the definition of blocks using a genetic map and a single value of δ. Future works could take a step further and locally define block boundaries in order to find the more relevant ones in terms of association [30, 31].

## Supplementary information

Supplementary Material
