# Supplementary material for: Genome-wide haplotype association study in imaging genetics using whole-brain sulcal openings of 16,304 UK Biobank subjects
Source: Eur J Hum Genet. 2021 Mar 4;29(9):1424–37. doi: 10.1038/s41431-021-00827-8 (PMC8440755; doi:10.1038/s41431-021-00827-8)
Supplement: Supplementary file 1 — Supplementary Material [file 41431_2021_827_MOESM1_ESM.pdf]

Supplementary Material 1 Box-Cox transformation of Sulcal Opening

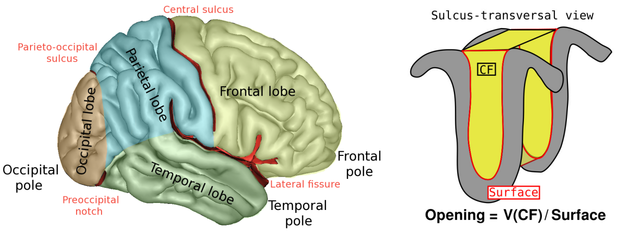

Supplementary Figure 1: (left) Main brain structures. Sulci are the main furrows. Here only two sulci are labelled. For example, Central Sulcus is separating Frontal and Parietal lobes (Blausen Medical Encyclopedia). (right) Sulcus opening, a measure of the average sulcus width, is computed as the ratio of the volume of Cerebrospinal Fluid to the surface of the sulcus.

For each subject, 123 labelled sulci were extracted from T1-weighted images. For each sulcus, a measure of its width - a feature called opening - is computed as the ratio of the volume of the Cerebrospinal Fluid the sulcus contains to its surface. For each sulcus, after adjusting for age and sex using linear regression, we identified and excluded outliers in the residual distribution using the robust interquartile range (IQR) method [18]. The distributions of sulcal opening values could exhibit deviation from the normal distribution. We evaluated these deviations and normalized them using a one-parameter Box-Cox transformation (power transformation) : Let $\mathbf{Y}$ and $\tilde{\mathbf{Y}}$ be the vectors of the initial and Box-Cox transformed sulcal opening values respectively. The one-parameter Box-Cox transformation is the following:

$$\tilde{\mathbf{Y}} = \begin{cases} \frac{\mathbf{Y}^{\lambda}-1}{\lambda} & if \quad \lambda \neq 0 \\ log(\mathbf{Y}) & if \quad \lambda = 0 \end{cases}$$

Supplementary Material 2 Haplotype count matrix

The following shows an example of Haplotype count matrix $\mathbf{H}$, obtained with 3 phased SNPs and 3 subjects: the matrix $\mathbf{H}$ accounts for the number of copies, per subject, of the two observed alternative haplotypes $h_1 = [101]$ and $h_2 = [010]$. In our example, subject S_1 is homozygous for haplotype h_0 and subjects S_2 and S_3 are heterozygous with haplotypes h_1 and h_2, and haplotypes h_0 and h_2 respectively.

$$\begin{array}{c} \\ S_{1,1} \\ S_{1,2} \\ S_{2,1} \\ S_{2,2} \\ S_{3,1} \\ S_{3,2} \end{array} \underbrace{\begin{array}{c} \begin{array}{ccc} Snp_1 & Snp_2 & Snp_3 \end{array} \\ \begin{bmatrix} 0 & 0 & 0 \\ 0 & 0 & 0 \\ 1 & 0 & 1 \\ 0 & 1 & 0 \\ 0 & 0 & 0 \\ 0 & 1 & 0 \end{bmatrix} \end{array}}_{\substack{\text{UK Biobank} \\ \text{haplotype dataset}}} \longrightarrow \begin{array}{c} \\ S_1 \\ S_2 \\ S_3 \end{array} \underbrace{\begin{array}{c} \begin{array}{ccc} h_0 & h_1 & h_2 \end{array} \\ \begin{bmatrix} 2 & 0 & 0 \\ 0 & 1 & 1 \\ 1 & 0 & 1 \end{bmatrix} \end{array}}_{\mathbf{H}} \qquad (3)$$

Supplementary Material 3 Univariate single-SNP test classically used in GWAS

The test for each of the p SNPs in classical GWAS for quantitative traits uses the following linear model :

$$\tilde{\mathbf{Y}} = \mathbf{X}\beta + \mathbf{s_j}\omega_j + \epsilon \qquad \text{for } 1 \leq j \leq p \tag{4}$$

with $\tilde{\mathbf{Y}}$ the phenotype vector, $\mathbf{X}$ the matrix of covariates and $\mathbf{s_j}$ the count vector of minor alleles for the SNP s_j, included with fixed effects β and ω_j respectively, and ϵ the error vector. Using PLINK, we computed a standard two-sided p-value of the t-statistic to test the following null hypothesis :

$$H_0 : \omega_j = 0 \qquad \text{vs} \qquad H_1 : \omega_j \neq 0$$

Supplementary Material 4 Length of haplotype blocks along the genetic map

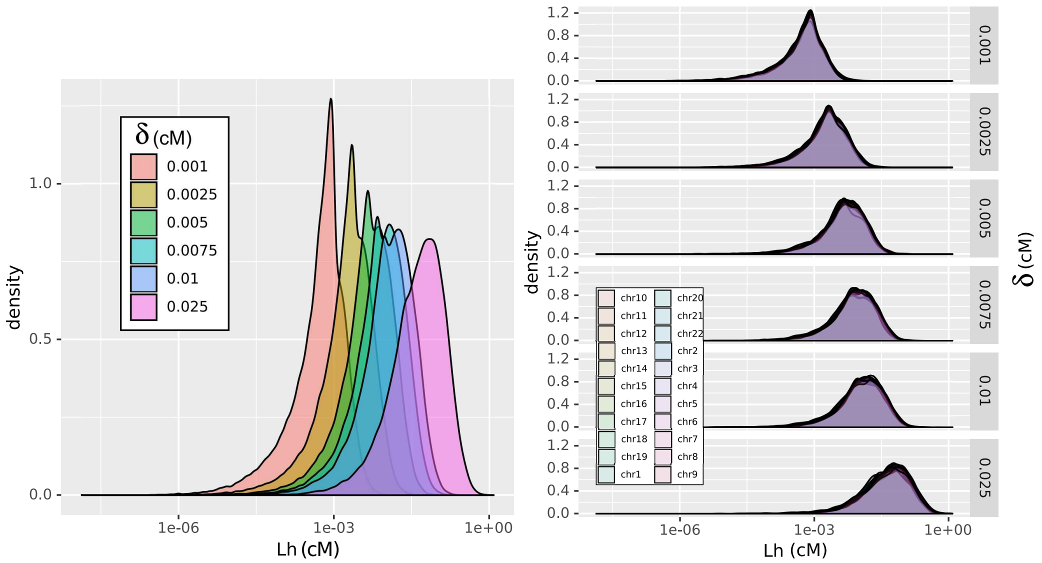

Supplementary Figure 2: Distribution of haplotype block length, in cM for increasing value of parameter δ

Supplementary Material 5 False Positive Rate under null hypothesis

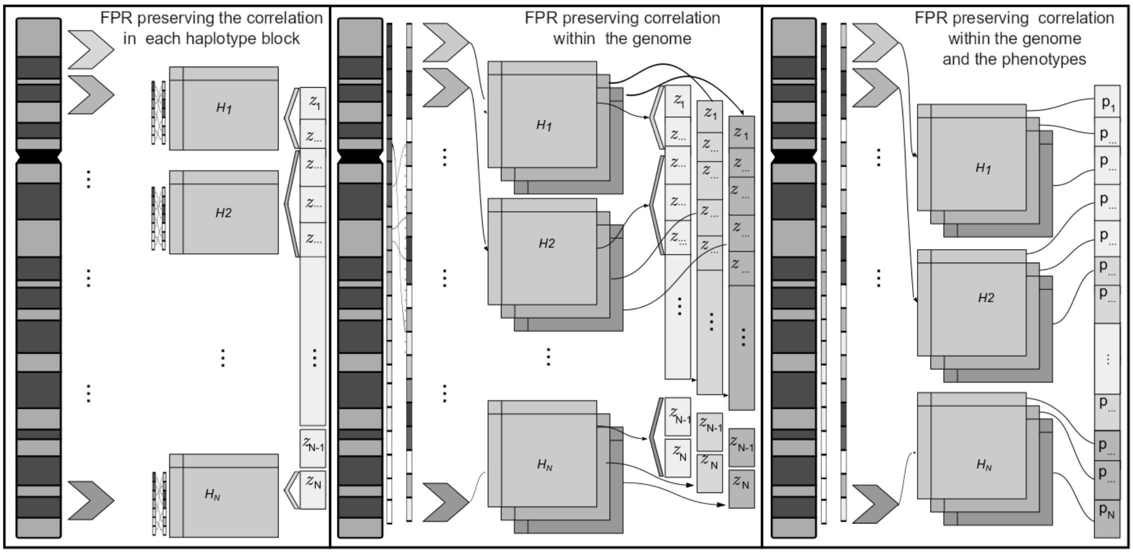

Supplementary Figure 3: Schematics of the 3 strategies described in 2.2 for determining FPR in genome-wide haplotype analysis. For each strategy, the chromosome map on the left represent the genome, and the arrows next to it the haplotype blocks. The Haplotype matrix for each block can be associated one phenotype (left panel) or with several, as represented by the multiple layers in middle and right panel. Permutations are represented by the reordering of the greyscale, for each block on the left panel, or for the whole genome (middle and right panel). On the middle panel, p-values for each phenotypes are studied separately (one p-value distribution for each phenotype)

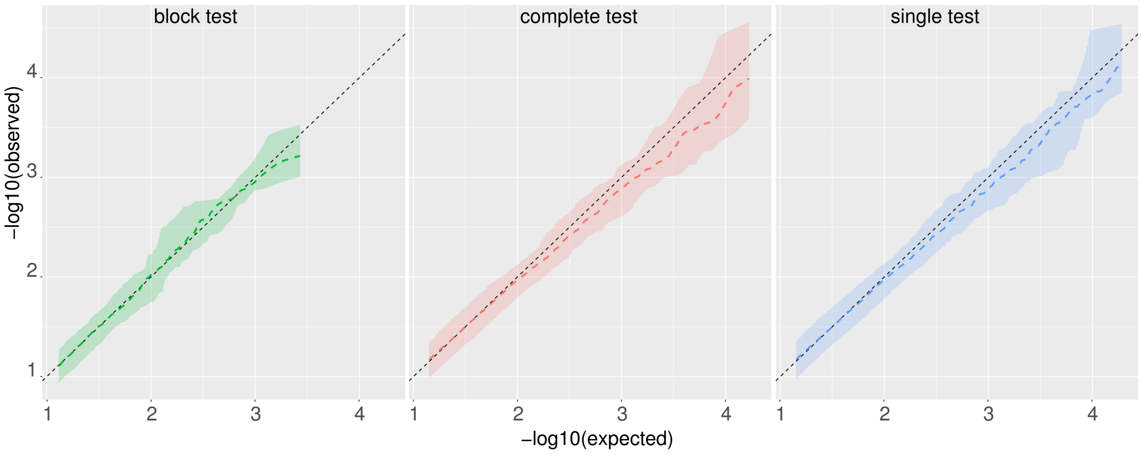

Supplementary Figure 4: Aggregated Q-Q plots under the null-hypothesis while preserving the correlation within the haplotype blocks (see section 2.3.5 first scenario, for details) using block-test, complete-test and single-test. We selected three phenotypes : two that represent the range of λ values (see 2.2 for details) and one for the most significant association found with the original dataset. For each test, we computed a Q-Q plot for each of the three phenotypes. The shaded area define the hull (minimum and maximum values) of the Q-Q plots for the 3 phenotypes and the average Q-Q plot is given by the coloured, dotted line. The black, dashed line at $y = x$ indicates where the expected distribution lies. For the three tests and the three phenotypes, the procedure did not produce any False Positive using $\alpha = 0.05$ and Bonferroni correction.

	block		complete		single			block		complete		single	
**Phenotypes**	**left**	**right**	**left**	**right**	**left**	**right**	**Phenotypes**	**left**	**right**	**left**	**right**	**left**	**right**
**FCLa**	0.00	0.00	0.00	0.00	0.04	0.00	**SFpolairetr**	**0.08**	0.04	**0.08**	0.00	**0.12**	0.04
**FCLp**	0.04	0.04	0.04	**0.08**	0.04	**0.08**	**SFsup**	0.00	**0.08**	0.04	0.04	0.04	0.04
**FCLrant**	0.00	**0.08**	0.00	0.00	0.00	0.00	**SGSM**	0.04		0.00		0.04	
**FCLrasc**	0.00	**0.08**	0.00	0.04	0.00	0.04	**SLiant**	**0.08**	0.00	**0.08**	0.00	0.04	0.00
**FCLrdiag**	0.04	**0.08**	0.00	0.00	0.00	0.04	**SLipost**	0.04	0.04	0.00	0.04	0.00	0.04
**FCLrretroCtr**	0.00	0.00	0.00	0.00	0.00	0.00	**SOlf**	0.04	0.00	0.00	0.04	0.00	0.04
**FCLrscant**	0.00	0.04	0.00	**0.08**	0.00	0.04	**SOp**	0.04	0.00	0.00	0.00	0.00	0.00
**FCLrscpost**	0.00	0.04	0.00	0.00	0.00	0.04	**SOr**	0.00	0.04	0.00	0.00	0.00	0.00
**FCMant**	**0.12**	0.04	0.00	0.04	0.00	0.04	**SOTlatant**	**0.12**	0.00	**0.08**	0.00	**0.08**	0.00
**FCMpost**	**0.08**	0.04	0.00	0.00	0.04	0.00	**SOTlatint**	0.00	0.04	0.00	0.00	0.00	0.00
**FColl**	0.04	**0.08**	0.04	0.00	0.04	0.00	**SOTlatmed**	0.04	0.00	0.04	**0.08**	0.00	**0.08**
**FIP**	0.00	0.00	0.00	0.00	0.00	0.04	**SOTlatpost**	**0.08**	**0.12**	0.04	**0.12**	0.04	**0.08**
**FIPPoCinf**	0.00	0.00	0.00	0.00	0.00	0.00	**SPaint**	0.04	**0.08**	0.04	0.04	0.04	0.00
**FIPrint1**	0.04	**0.12**	0.00	**0.08**	0.04	0.04	**SPasup**	0.00	0.04	0.00	0.00	0.00	0.00
**FIPrint2**	0.04	0.04	0.04	0.00	0.04	0.00	**SPat**	**0.08**	0.00	0.00	0.00	0.00	0.00
**FPO**	0.00	0.00	0.00	0.04	0.00	0.04	**SpC**	0.04	0.00	0.00	0.00	0.00	0.00
**INSULA**	0.04	0.00	0.00	0.04	0.00	0.00	**SPeCinf**	0.00	**0.08**	**0.08**	**0.12**	**0.08**	**0.08**
**OCCIPITAL**	**0.20**	0.04	0.04	0.00	**0.08**	0.00	**SPeCinter**	0.04	0.00	0.00	0.04	0.00	0.04
**SC**	0.00	0.04	0.04	0.04	0.00	0.00	**SPeCmarginal**	0.04	0.00	0.00	0.00	0.00	0.00
**SCall**	**0.12**	**0.12**	0.04	0.00	0.04	0.04	**SPeCmedian**	0.00	0.00	0.00	0.00	0.00	0.04
ScCal.FCalant	***0.32***	0.00	***1.00***	**0.08**	***1.00***	**0.08**	**SPeCsup**	0.00	**0.12**	**0.08**	0.00	0.04	0.04
**SCLPC**	0.00	0.00	0.04	0.00	0.04	0.04	**SPoCsup**	0.04	0.04	**0.08**	0.00	**0.08**	0.04
**SCsylvian**	0.00	0.00	0.00	0.00	0.00	0.04	***SRh***	***0.64***	0.04	***1.00***	**0.28**	***1.00***	**0.28**
**SCu**	**0.08**	0.00	0.00	0.04	0.00	0.04	**SRinf**	0.00	0.00	0.00	0.00	0.04	0.00
**SFinf**	0.00	0.00	0.04	0.00	0.04	0.00	**SsP**	0.00	**0.08**	**0.08**	**0.12**	**0.12**	**0.08**
**SFinfant**	0.04	0.00	0.00	0.00	0.00	0.00	**STiant**	**0.08**	0.00	0.04	0.00	0.04	0.00
**SFint**	0.04	0.04	0.04	0.00	0.04	0.00	**STipost**	**0.08**	0.04	0.00	0.04	**0.12**	0.04
**SFinter**	0.04	0.00	0.04	0.00	0.04	0.00	**STpol**	0.00	0.00	0.04	0.00	0.00	0.04
**SFmarginal**	0.00	0.00	0.00	0.00	0.00	0.00	**STs**	**0.20**	0.04	**0.12**	0.00	**0.16**	0.00
**SFmedian**	0.00	0.04	**0.08**	**0.12**	0.00	**0.08**	**STsterascant**	**0.08**	**0.08**	0.04	0.04	**0.08**	0.00
**SForbitaire**	0.04	0.00	**0.08**	0.00	**0.08**	0.04	**STsterascpost**	0.00	0.00	0.00	0.00	0.00	0.00

Supplementary Table 1: Study of the False Positive Rate under the null hypothesis for 25 runs of permutation using the second scenario (see section 2.3.5 for details). P-values are corrected using Bonferroni for N_T hypotheses, and discovery threshold set to $\alpha = 0.05$ (see 2.3.4). The table shows the proportion of runs where we observed inflation of FPR (e.g., FPR$\geq 1/N_T$.), with, in bold, the proportion > 0.05 When we pool the FPR results of all permuted phenotypes, we seem to control the family-wise error rate at 5% under the global null hypothesis for each of the three tests (block test :0.041, complete test : 0.043 and single test: 0.046). In italic, two phenotypes raise concern regarding inflation of FPR for all 3 tests : Fcalant.ScCal_right and SRh_left but were not found significantly associated with any haplotype in the real dataset.

Test	Average number of test per run	Average discovery threshold α'=α/NT	Average FPR per run	FPR over all runs	# of runs	# of runs with FPR≥1/NT	proportion of run with FPR≥1/NT	Min FPR	Max FPR
block	14,700,330	3.40E-09	2.72E-09	0.040	25	1	0.040	0	6.80E-08
complete	110,575,583	4.52E-10	1.81E-09	0.200	25	5	0.200	0	9.06E-09
single	124,659,108	4.01E-10	9.59E-10	0.120	25	3	0.120	0	8.00E-09
Phenotypes with >75% individuals observed									
block	13,505,181	3.70E-09	2.96E-09	0.040	25	1	0.040	0	7.40E-08
complete	100,190,899	4.99E-10	2.80E-09	0.280	25	6	0.240	0	2.00E-08
single	114,524,221	4.37E-10	1.04E-09	0.120	25	3	0.120	0	8.70E-09
Phenotypes with >75% individuals observed, excluding outliers									
block	13,266,152	3.77E-09	3.02E-09	0.040	25	1	0.040	0	7.54E-08
complete	98,507,148	5.08E-10	1.63E-09	0.160	25	4	0.160	0	1.02E-08
single	112,497,243	4.45E-10	3.54E-10	0.040	25	1	0.040	0	8.86E-09

Supplementary Table 2: Study of the False Positive Rate under the null hypothesis for 25 runs of permutation using the third scenario (see section 2.3.5 for details). P-values are corrected using Bonferroni for N_T hypotheses, and discovery threshold set to $\alpha = 0.05$ (see 2.3.4). Top table shows FPR for all phenotypes on 25 permutations. Middle table shows FPR for phenotypes that were observed in more than 75% of the individuals. Bottom table shows FPR for phenotypes that were observed in more than 75% of the individuals with the exclusion of the two phenotypes showing systematic inflation of FPR (Fcalant.ScCal_right and SRh_left).

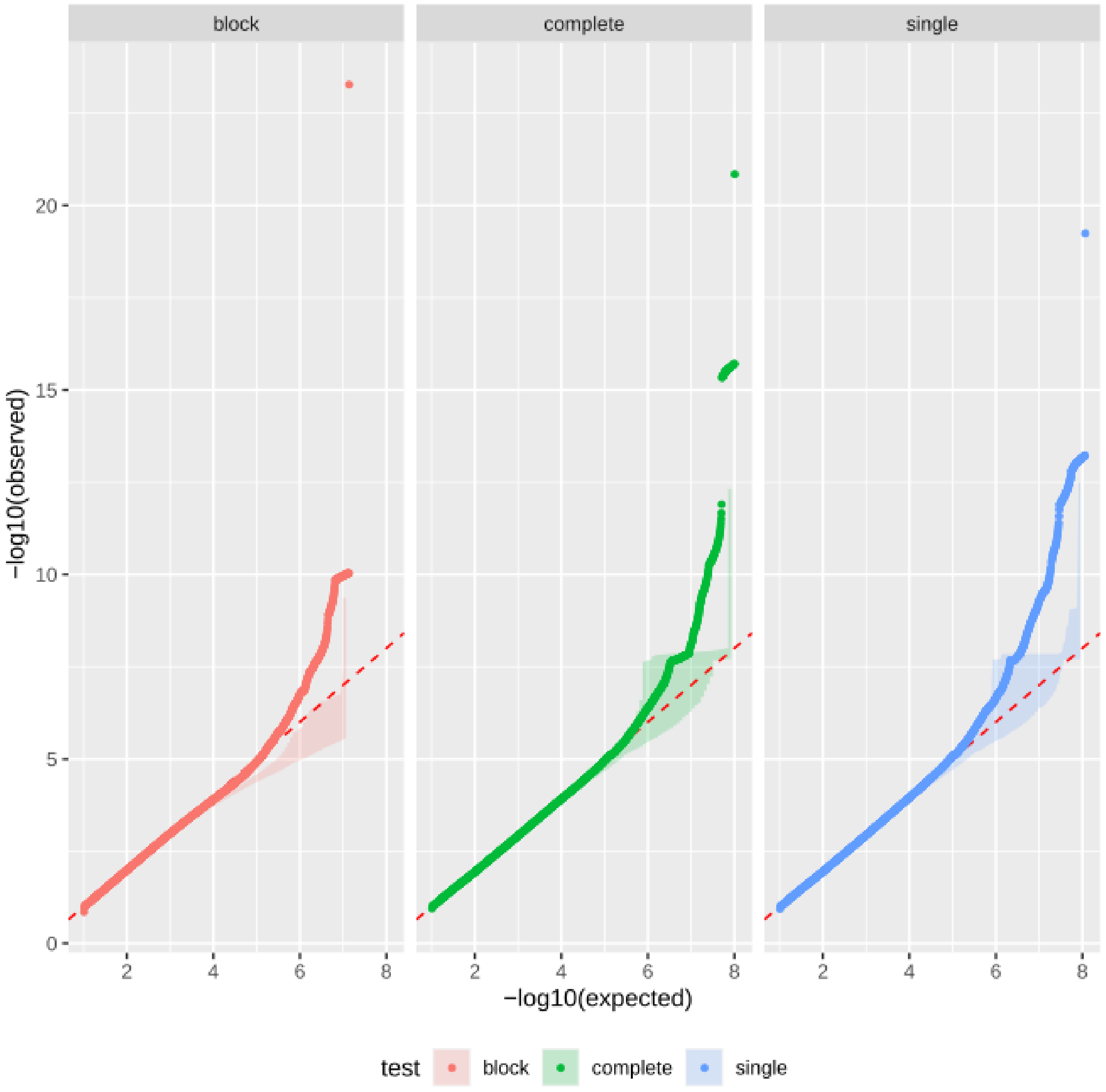

Supplementary Figure 5: Aggregated Q-Q plots for phenotypes that were observed in more than 75% of the individuals. For each test and each permutation under the null-hypothesis while preserving the correlation within the genomes and the phenotypes (see section 2.3.5 ; third scenario), we computed a Q-Q plot and the shaded area define the hull (minimum and maximum values) of the Q-Q plots.The Q-Q plot for the real data is given by the coloured, dotted line. The red, dashed line at $y = x$ indicates where the expected distribution lies.

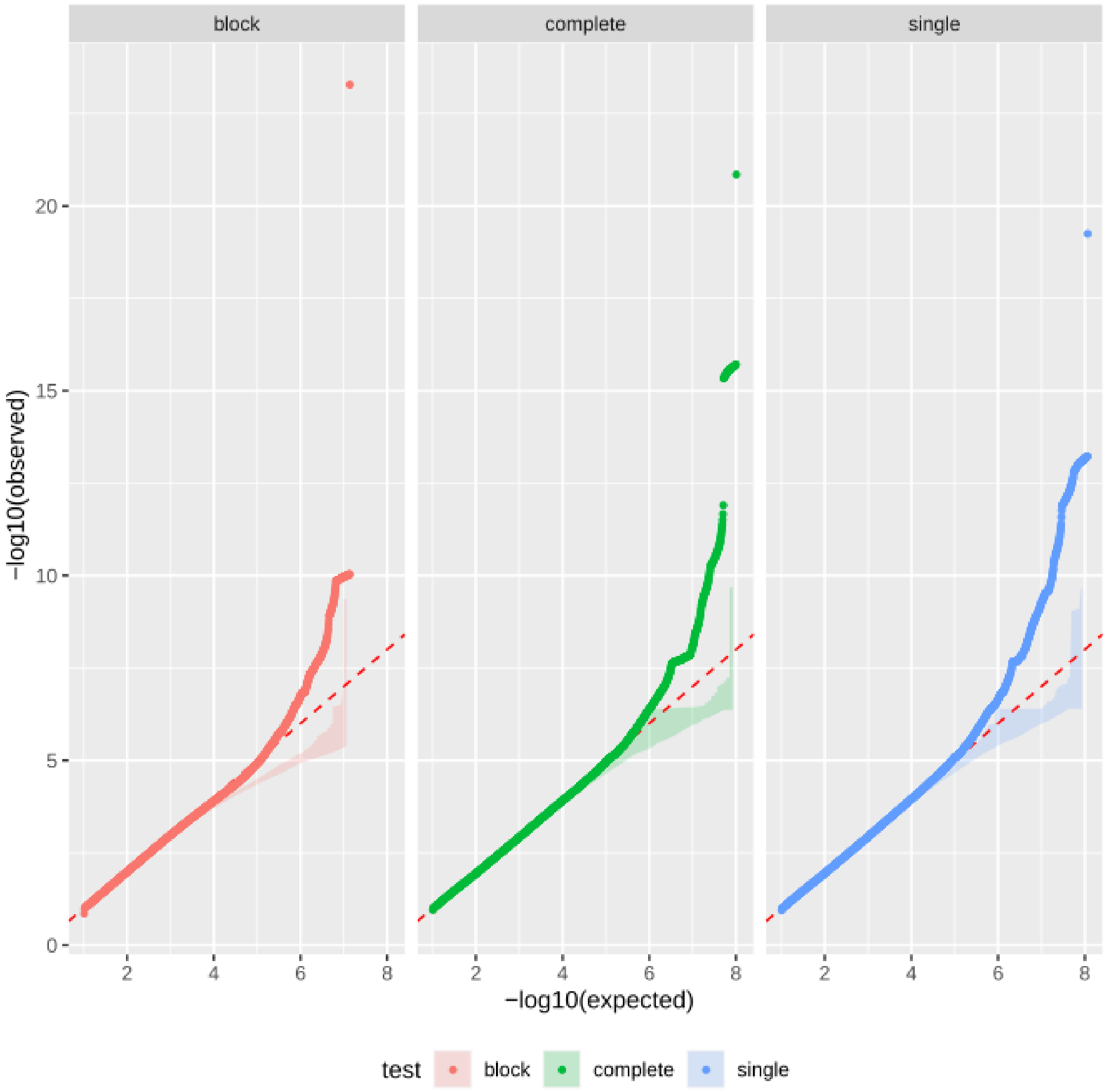

Supplementary Figure 6: Aggregated Q-Q plots for phenotypes that were observed in more than 75% of the individuals and after exclusion of two phenotypes that showed systematic inflation of FPR (Fcalant.ScCal_right and SRh_left) in Supplementary Table 1. For each test and each permutation under the null-hypothesis while preserving the correlation within the genomes and the phenotypes (see section 2.3.5 ; third scenario), we computed a Q-Q plot and the shaded area define the hull (minimum and maximum values) of the Q-Q plots.The Q-Q plot for the real data is given by the coloured, dotted line. The red, dashed line at $y = x$ indicates where the expected distribution lies.

Supplementary Material 6 Tissue expression of gene related to significant hits

The hit located on chr.7q is found only associated to Insula (left and right) openning. The insula is a cortical region known to integrates emotional, cognitive, and motivational signals. Haplotypes of the block chr7:13441632-134416604 are located in a genomic region comprising several genes and markers associated with Autism Spectrum disorders (see Table 1). This hit could carry a signal distinct from the others because it is the only single phenotype (bilateral) - single haplotype block significant association.

Haplotype found on chr9:113 are located within an intronic region of LPAR1 gene, which is encoding for a protein used in cell signalling, notably in inhibition of neuroblastoma cell differentiation. Stankoff *et al.* [33] showed that transcripts are not detected during early stages of oligodendroglial development, but are expressed only in mature oligodendrocytes, shortly before the onset of myelination. Transcript are expressed in different brain tissues in GTEx v8 (see Fig 7)

Haplotypes in block on chr.12:106476140-106477376 are located within the gene NUAK1, covering large intronic parts as well as 2 small exons. NUAK1 codes for a protein involved in cell proliferation, for example in arborization of mammalian neurons, regulating axon branching [32]. Particularly, it is expressed in several brain tissues (see Fig. 8).

Haplotypes found in the chr16:87Mbp region are associated with several sulci located on both sides of the frontal region, in particular the Inferior Frontal Sulcus (SFinf). Located within gene C16orf95, haplotypes are 100kbp upstream of RP11-178L8.8 (or AC010531.7), a non-coding lncRNA mostly expressed in the brain (GTEx v6). This locus is antisens to FXBO31, a gene predominently expressed in Brain tissues in GTEx v8 (see Fig. 9).

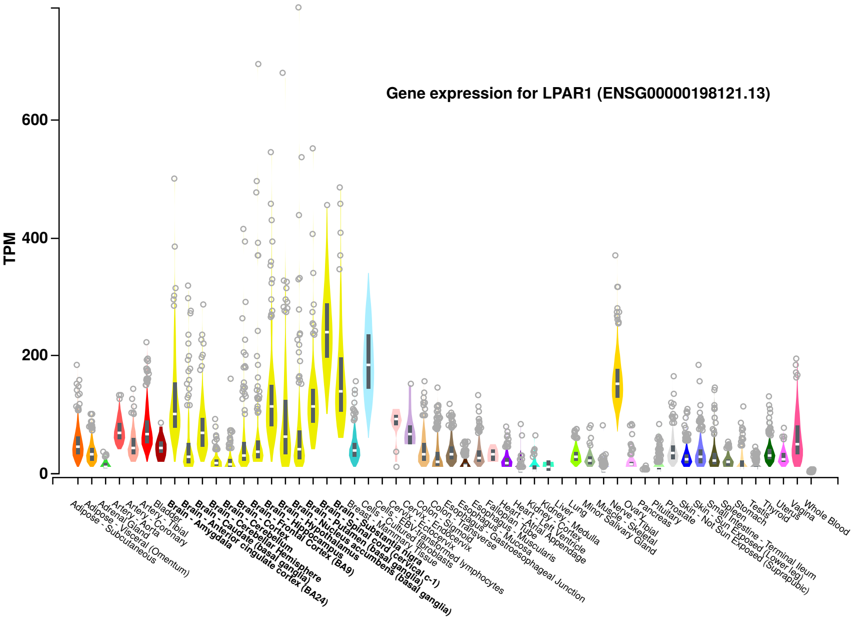

Supplementary Figure 7: Expression of LPAR1 gene in different tissues of the brain (GTEx v8), related to top hit on chromosome 9.

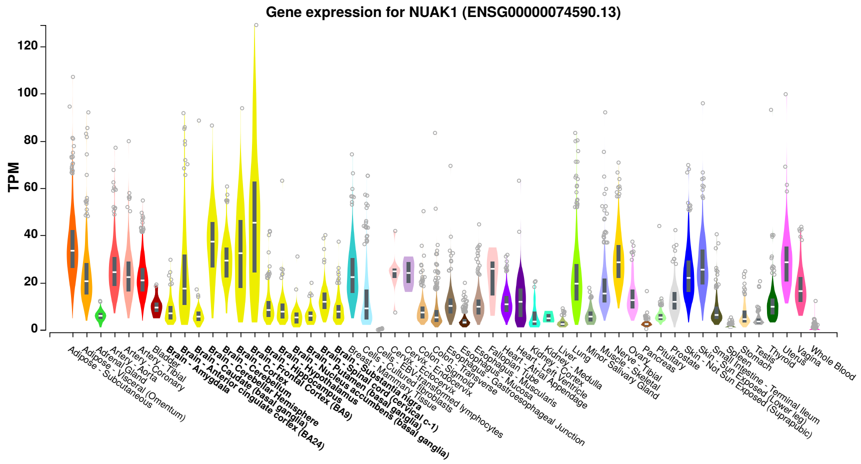

Supplementary Figure 8: Expression of NUAK1gene in different tissues of the brain (GTEx v8), related to top hit for chromosome 12.

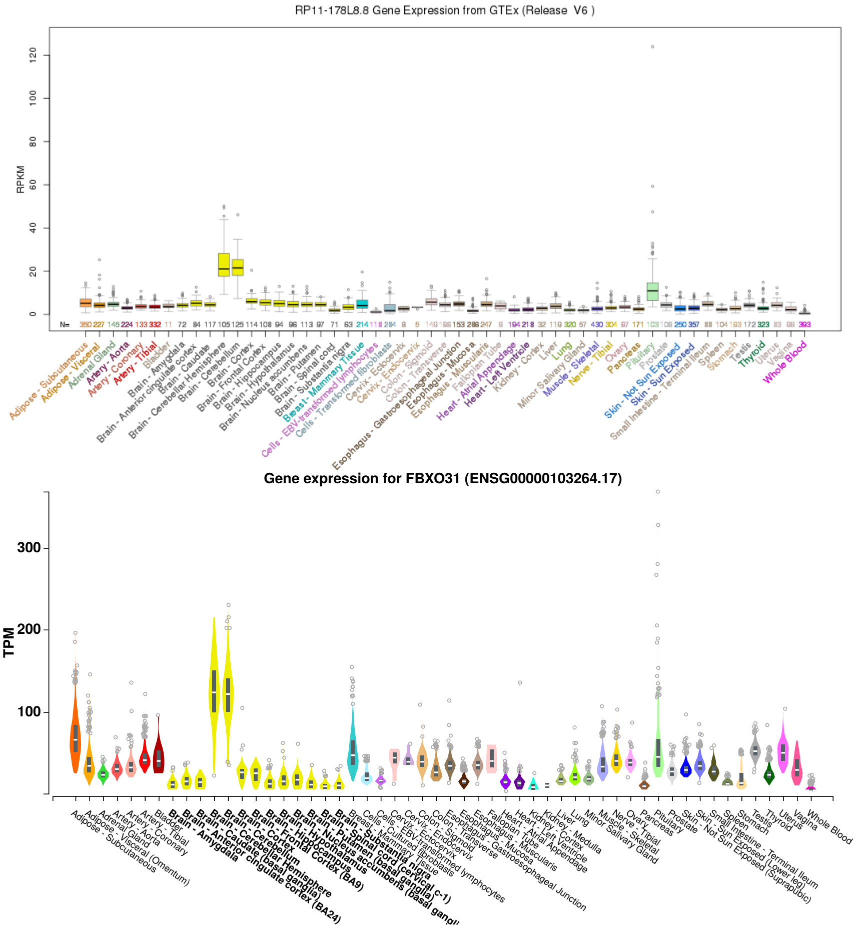

Supplementary Figure 9: (Top) Expression of antisens lrcRNA RP11-178L8.8 in different tissues of the brain (GTEx v6), 102kbp downstream of top hit for chromosome 16 (Bottom) Expression of FBXO31 gene in different tissues of the brain (GTEx v8), 102kbp upstream of top hit for chromosome 16.

Supplementary Material 7 Manhattan plots for single-SNPs associations

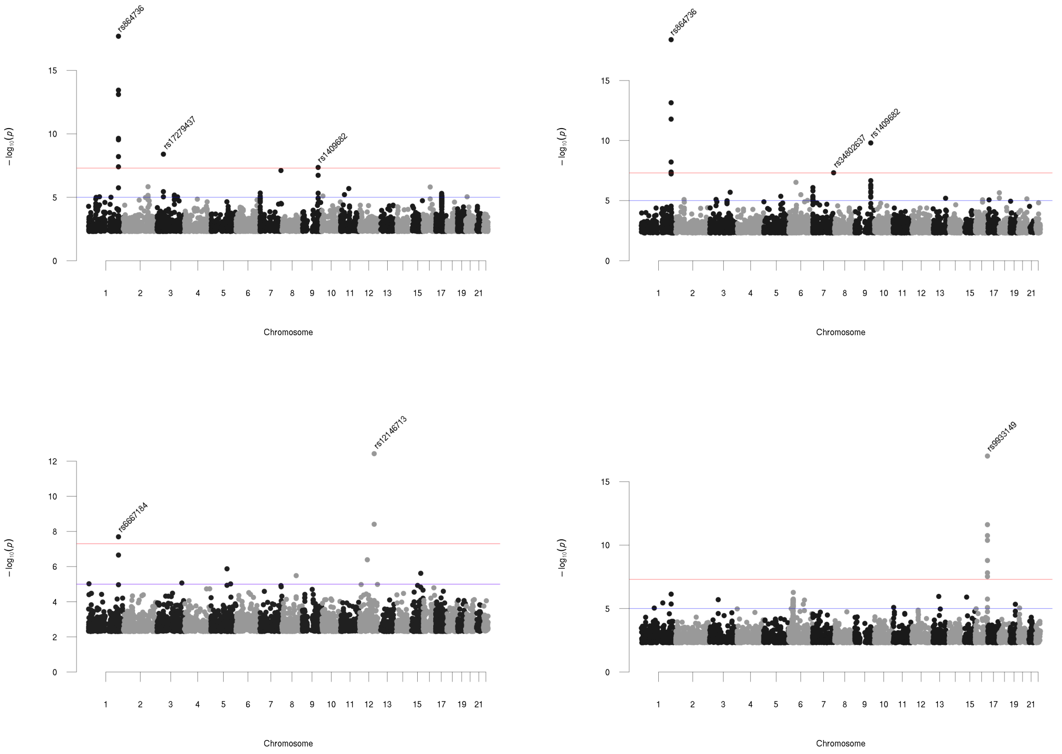

Supplementary Figure 10: Manhattan plot for single SNP associations (genotyped SNPs) with FCMpost Left(chr1, top left), FCMpost Right (chr9, top right), SFint. Right (chr12, bottom left), SFinfant. Right (chr16, bottom right)

Supplementary Material 8 Manhattan plots for haplotype associations

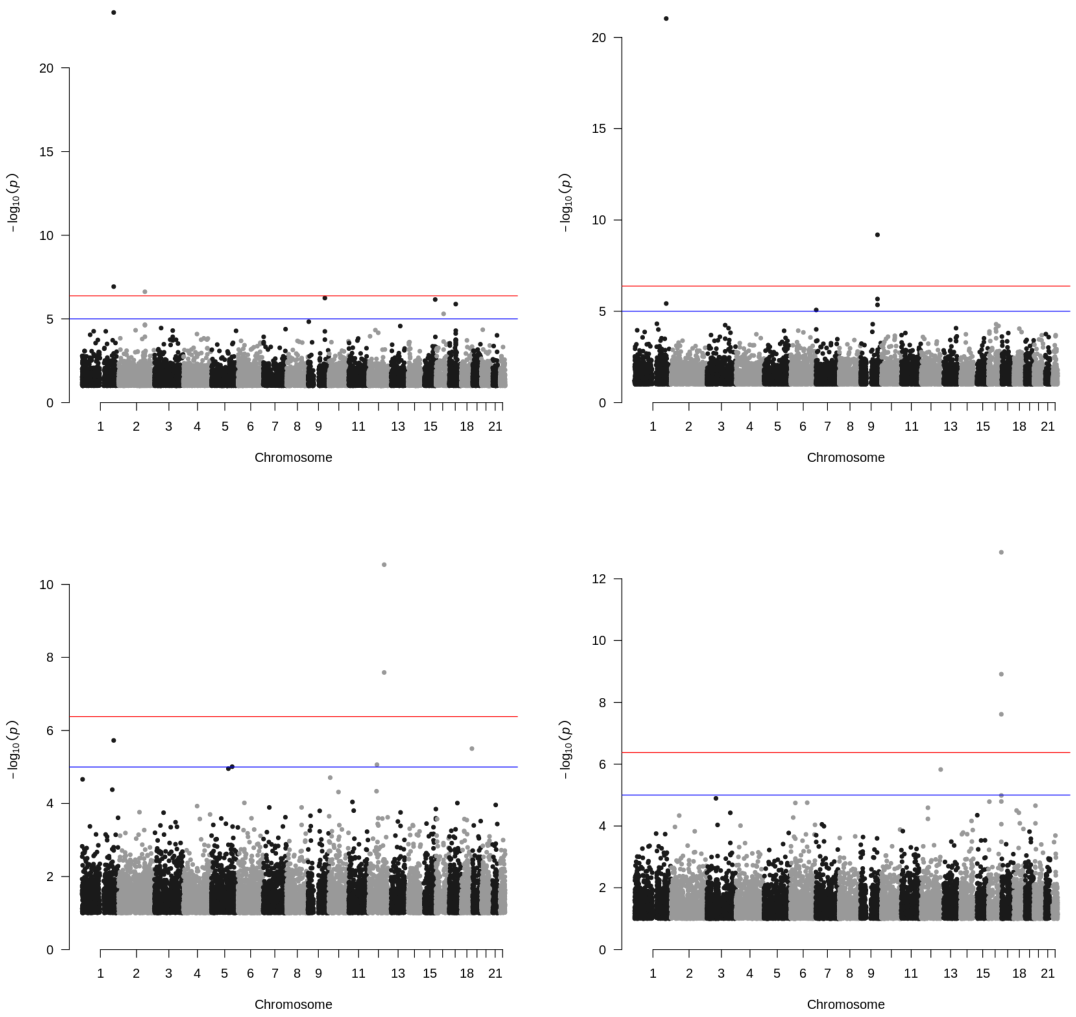

Supplementary Figure 11: Manhattan plot for haplotype associations (block test) with FCMpost Left(chr1, top left), FCMpost Right (chr9, top right), SFint. Right (chr12, bottom left), SFinfant. Right (chr16, bottom right)

Supplementary Material 9 Sulci of which openning is significantly associated with at least one genomic region

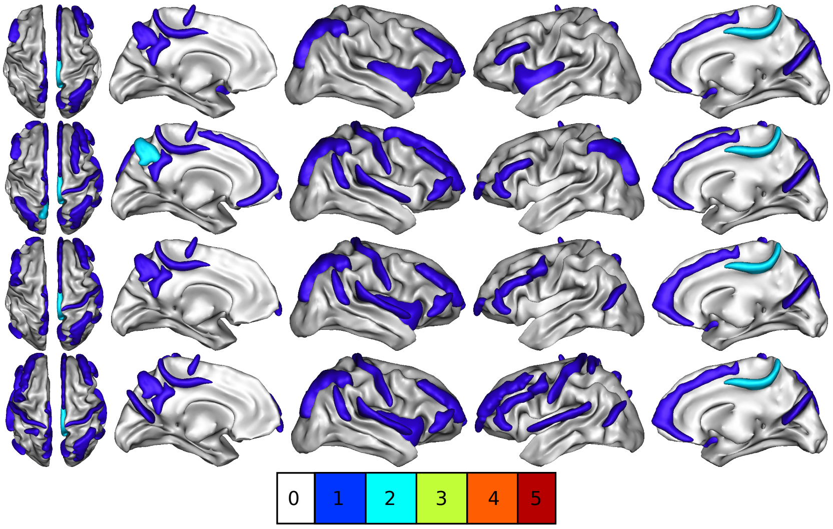

Supplementary Figure 12: Brain sulci coloured according to the number of significant hits. Hits - genomic region of 1 Mbp with at least one significant p-value among the three tests. Sulci colored in dark blue color are associated with one hit, light blue are associated with 2 hits (chr1:215 and chr9:114). In rows, from top to bottom, hits are determined using block-test, complete-test, single-test and imputed SNPs with PLINK..

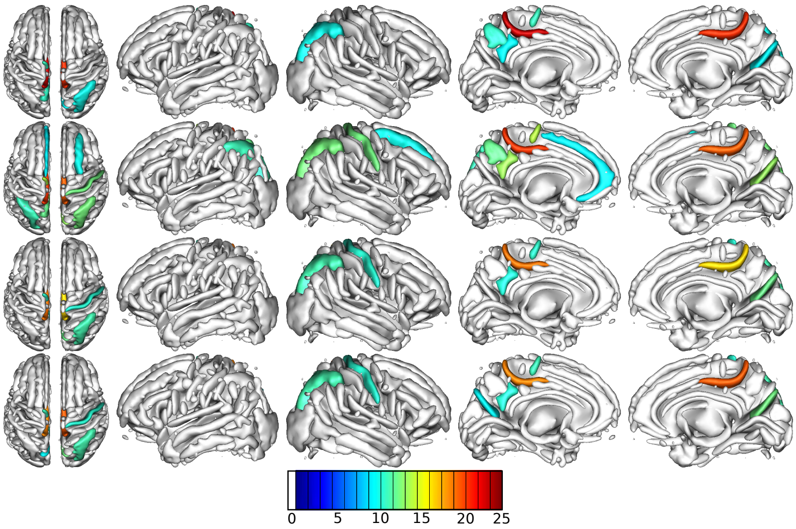

Supplementary Figure 13: Brain sulci coloured according to $-log10(p)$ of significant hits in the chr1:215 region. Hits - genomic region of 1 Mpb with at least one significant p-value among the three tests. In rows, from top to bottom, hits are determined using block-test, complete-test, single-test, imputed SNPs with PLINK.

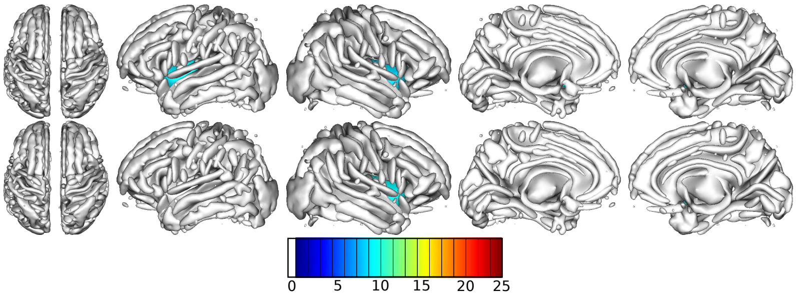

Supplementary Figure 14: Brain sulci coloured according to $-log10(p)$ of significant hits in the chr7:134 region. Hits - genomic region of 1 Mpb with at least one significant p-value among the three tests. In rows, from top to bottom, hits are determined using block-test and single-test.

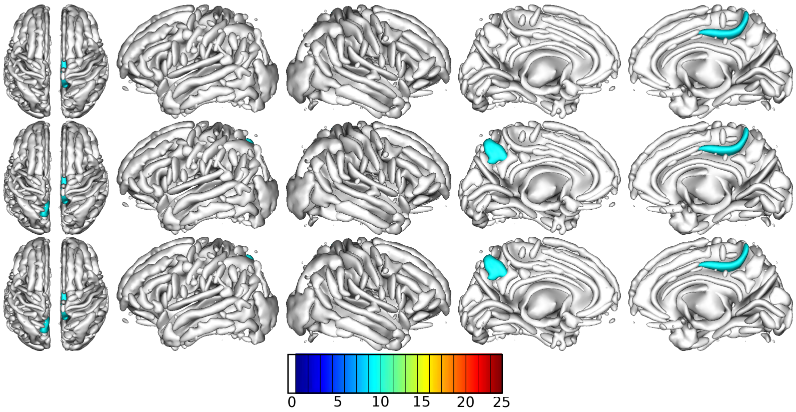

Supplementary Figure 15: Brain sulci coloured according to $-log10(p)$ of significant hits in the chr9:114 region. Hits - genomic region of 1 Mpb with at least one significant p-value among the three tests. In rows, from top to bottom, hits are determined using block-test, complete-test, single-test

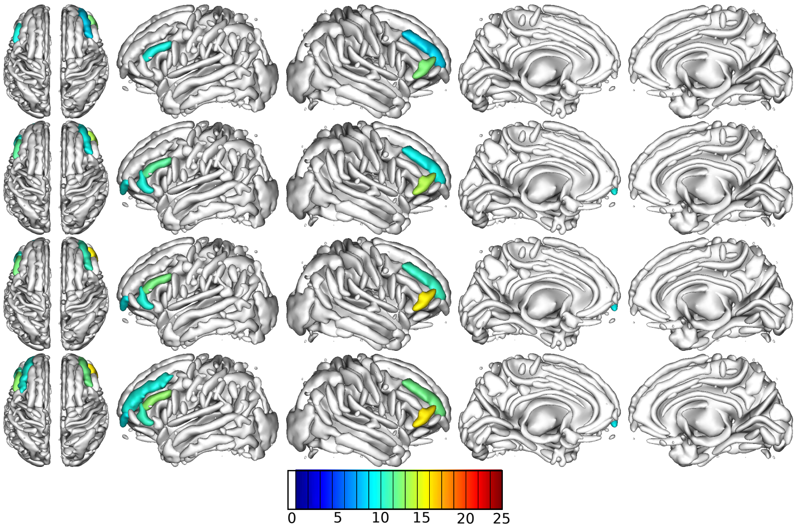

Supplementary Figure 16: Brain sulci coloured according to $-log10(p)$ of significant hits in the chr16:87 region. Hits - genomic region of 1 Mpb with at least one significant p-value among the three tests. In rows, from top to bottom, hits are determined using block-test, complete-test and single-test.
